# Supplementary material for: Automated Machine Learning (AutoML)-Derived Preconception Predictive Risk Model to Guide Early Intervention for Gestational Diabetes Mellitus
Source: Int J Environ Res Public Health. 2022 Jun 1;19(11):6792. doi: 10.3390/ijerph19116792 (PMC9180245; doi:10.3390/ijerph19116792)
Supplement: Supplementary file 1 [file ijerph-19-06792-s001.zip › Supplementary Material File S1_20220601.pdf]

Supplementary Material

Introduction to Genetic Programming

Genetic programming (GP) is a branch of evolutionary algorithm, a subset of machine learning. Inspired by the Darwinian principles of natural selection, GP is the programming of computers by natural selection. Supplementary Figure 1 illustrates the GP loop, where a new offspring is created by either mutation or crossover [25]. In this population ensemble method, the reproductive success of individuals depends on how well they adapt to the environment relative to the rest of the population.

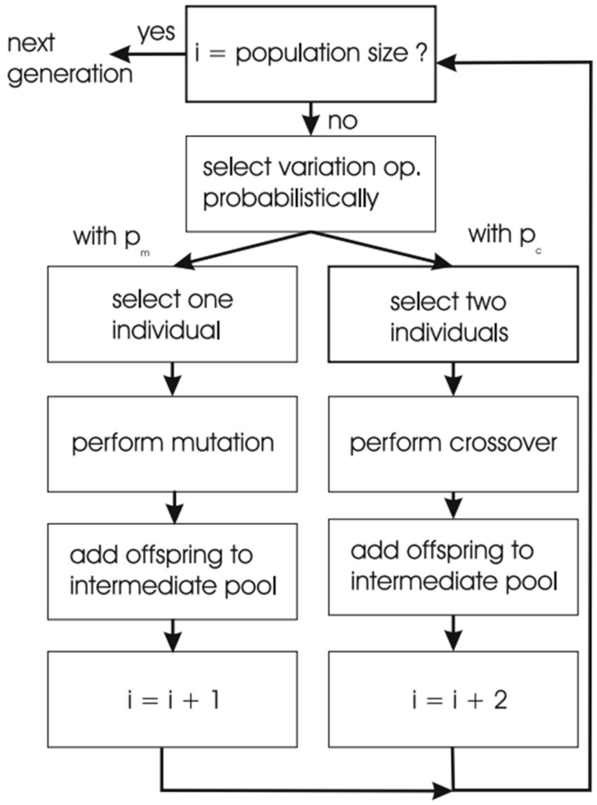

Supplementary Figure S1. GP Loop. GP flowchart describes the options for filling the intermediary population in a generational scheme.

Modelling problems are special cases of optimization and GP seek models with maximum fit. The data structure used to encode candidate solutions in GP are in the form of tree representation (models are represented as parse trees) to optimize the modelling operations on the dataset. Models are treated as individuals (whole chromosomes) and their fitness is the model quality to be maximized [26].

GP processes a whole collection of candidate solutions simultaneously by creating an ensemble out of the populations. In the context of Automated Machine Learning (AutoML), the automatic evolution of computer programs are used to generate optimal solutions of models and hyperparameters. Tree nodes in TPOT comprises of operators: preprocessors, decomposition functions, feature selectors or classifiers. Identical copies of input data enter the tree at leaf nodes and predictions are output at root node [28]. The stacking estimator component in TPOT allows classification models to propagate the outputs as synthetic features to subsequent operators. The parameters of operators are optimized during the training process.

With the default TPOT settings, 100 generations with 100 population sizes, TPOT evaluated 10,000 pipelines for optimal classification performance. With 5-fold stratified cross-validation, TPOT evaluated 50,000 different pipelines before completing the evolutionary search process. Similar to natural evolution, the fitter architectures were propagated forward to future generations, while the less fit architectures died out (survival of the fittest).

The GP algorithm in TPOT have the following general properties:

(1) Selection - A population of possible solutions and the fitness function (classification performance). At every iteration, each of the fitted pipeline is evaluated. 100 individuals were retained in the GP population of every generation.

(2) Crossover - The selection of best (fittest) solution and performing crossover to create a new population. Crossover rate (default = 0.1) instructs the GP algorithm on how many pipelines to breed every generation (exchange of subtrees). The number of offsprings to produce in each GP generation was set the same as population size (default = 100).

(3) Mutation - The offspring from previous step are mutated with some random modifications until the best solution is reached. Mutation rate (default = 0.9) instructs the GP algorithm on how many pipelines to apply random changes to every generation (random change in trees).

The exported AutoML pipeline for the best predictive model is as follows (Python code snippet with hyperparameters):

```
exported_pipeline = make_pipeline(
    PolynomialFeatures(degree=2, include_bias=False, interaction_only=False),
    StackingEstimator(estimator=SGDClassifier(alpha=0.01, eta0=0.1, fit_intercept=True, l1_ratio=0.75, learning_rate="invscaling", loss="modified_huber", penalty="elasticnet", power_t=0.1)),
    GradientBoostingClassifier(learning_rate=0.5, max_depth=6, max_features=0.1, min_samples_leaf=1, min_samples_split=17, n_estimators=100, subsample=1.0)
)
```
