# Supplementary material for: Automated Machine Learning (AutoML)-Derived Preconception Predictive Risk Model to Guide Early Intervention for Gestational Diabetes Mellitus
Source: Int J Environ Res Public Health. 2022 Jun 1;19(11):6792. doi: 10.3390/ijerph19116792 (PMC9180245; doi:10.3390/ijerph19116792)
Supplement: Supplementary file 1 [file ijerph-19-06792-s001.zip › Supplementary Material Table S1_20220601.pdf]

Supplementary Table S1. Sensitivity analysis of preconception predictive risk model.

Features	Optimal Machine Learning Pipeline	AUC
1: Fatty liver index + mean arterial blood pressure + fasting insulin + TG/HDL ratio	Stacked ensemble model with gradient boosting classifier, gaussian Naïve Bayes classifier, multinomial Naïve Bayes classifier, decision tree classifier and XGBoost classifier.	0.92
**2: HbA_{1c} + mean arterial blood pressure + fasting insulin + TG/HDL ratio**	Stacked ensemble model with gradient boosting classifier and linear support vector machine classifier (stochastic gradient descent training).	**0.93**
3: HbA_{1c} + fatty liver index + fasting insulin + TG/HDL ratio	Stacked ensemble model with random forest classifier, bernoulli Naïve Bayes classifier, gaussian Naïve Bayes classifier and multinomial Naïve Bayes classifier.	0.82
4: HbA_{1c} + fatty liver index + mean arterial blood pressure + TG/HDL ratio	Stacked ensemble model with random forest classifier and linear support vector machine classifier (stochastic gradient descent training).	0.89
5: HbA_{1c} + fatty liver index + mean arterial blood pressure + fasting insulin	Stacked ensemble model with multinomial Naïve Bayes classifier, multi-layer perceptron classifier, linear support vector machine classifier and XGBoost classifier.	0.86
6: Fasting glucose + mean arterial blood pressure + fasting insulin + TG/HDL ratio	Stacked ensemble model with random forest classifier, multinomial Naïve Bayes classifier and XGBoost classifier.	0.87
7: HbA_{1c} + systolic blood pressure + fasting insulin + TG/HDL ratio	Stacked ensemble model with gradient boosting classifier and gaussian Naïve Bayes classifier.	0.91
8: HbA_{1c} + mean arterial blood pressure + HOMA-IR + TG/HDL ratio	Gradient boosting classifier.	0.91

Note: Sensitivity analyses of preconception predictive risk model for GDM. The optimal machine learning pipeline for each model and area under the receiver operating characteristic curve (AUC) performance metric are reported.
